# Chemical recycling of polystyrene waste: theoretical-experimental study of the mechanism of catalytic transformation

**DOI:** 10.1039/d6ra01516f

**Published:** 2026-04-28

**Authors:** Dana Yanes Quintana, Victoria Colombo, María Estela Pronsato, Nilda Chasvin, Carolina Pistonesi, Alejandra Diez

**Affiliations:** a Departamento de Química, Universidad Nacional del Sur, INQUISUR, UNS-CONICET Av. Alem 1253 (8000) Bahía Blanca Argentina alejandra.diez@uns.edu.ar; b Departamento de Física, Universidad Nacional del Sur & IFISUR (UNS-CONICET) Av. Alem 1253 (8000) Bahía Blanca Argentina; c Facultad de Ciencias Exactas y Naturales, Universidad Nacional de la Pampa Santa Rosa Argentina

## Abstract

This study combines experimental and theoretical approaches to investigate the catalytic pyrolysis of polystyrene (PS) waste using CeO_2_ and Co/CeO_2_ catalysts. A laboratory-scale reactor was designed and optimized at 450 °C under a nitrogen atmosphere to maximize liquid product yield. The catalysts, synthesized *via* the combustion method and characterized by XRD, BET, and potentiometric titration, exhibited high surface areas (110 and 100 m^2^ g^−1^, respectively). Experimental results revealed that pure CeO_2_ selectively promoted PS depolymerization toward styrene monomer formation through a β-scission mechanism, achieving 87.04% styrene selectivity. In contrast, cobalt incorporation altered the reaction pathway, reducing styrene yield but increasing overall liquid fraction and calorific value, indicating a more energy-efficient process. Density functional theory (DFT) calculations supported these findings, showing that styrene dimer adsorption and β-scission on the CeO_2_(111) surface are energetically favorable, whereas Co modification raises the activation barrier and enhances dimer adsorption, suggesting a possible reduction in the accessibility of the catalyst's acid active sites. These combined results suggest that CeO_2_ is well-suited for selective monomer recovery, while Co/CeO_2_ offers a balanced route for both material and energy valorization of PS waste, thus advancing the development of catalytic systems for sustainable chemical recycling.

## Abstract 2

This study combines experimental and theoretical approaches to investigate the catalytic pyrolysis of polystyrene (PS) waste using CeO_2_ and Co/CeO_2_ catalysts.

## Graphical abstract

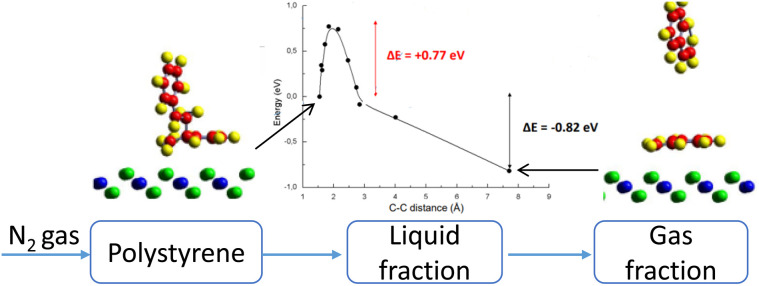

## Introduction

The successive socio-economic transformations and environmental concerns of recent decades have highlighted the need for sustainable processes that reduce and optimize society's waste. Global annual plastic production reached nearly 350 million metric tons in 2017 and continues to grow. Among the various uses of plastic, approximately 40% of total demand is for single use packaging. With the increasing demand for plastic, there is also a global rise in plastic waste generation. Only 9% of global plastic waste is recycled, while the remaining 91% ends up in landfills, is incinerated, or accumulates in the environment.^1,2^ In 2018, Argentina recovered 251 000 tons of plastic waste, of which 241 000 tons were recycled mechanically, and 10 000 tons were used for energy recovery in cement kilns. This represents 26% of total recycling and valorization for packaging, containers, and other plastic materials. However, the plastic recycling industry still operates with 60% idle capacity. Over the past 20 years, the circular economy for plastics in Argentina has evolved significantly, moving from a linear model of production, consumption, and disposal to a circular paradigm. This transition emphasizes responsible consumption, eco-design, recycling, education, and the adoption of the 7R framework (reduce, reuse, recycle, recover, redesign, remanufacture, and renew). Plastic recycling in Argentina has multiplied fivefold since 2003, with over 294 000 tons recycled in 2023.^3^ Recycling plastic waste is a significant global concern, and it is typically achieved through four main methods. Primary recycling involves closed-loop processes where uncontaminated plastic waste is recycled directly at the manufacturing site. Secondary recycling focuses on the mechanical processing of post-consumer plastic waste. Tertiary recycling uses chemical methods to break down plastics into their original chemical components, while quaternary recycling involves incineration to recover energy from plastic waste.^2^

The limitations of mechanical recycling, such as the need for homogeneous and contaminant-free materials, reduce its effectiveness and make it necessary to explore alternative methods.

Chemical recycling, especially through pyrolysis, emerges as a promising solution to convert plastic waste into high-value products, such as fuels (jet fuel, diesel), gases, and aromatic compounds. This process offers significant advantages over mechanical recycling, as it can handle heterogeneous mixtures and multilayer materials that are difficult to process. Additionally, the use of catalysts optimizes the yield and quality of the products by enabling efficient operation at lower temperatures.^2^

In recent years, polymer recycling strategies have evolved toward two complementary paradigms: molecular design for recyclability and thermochemical conversion. On one hand, advances in polymer chemistry have demonstrated that the incorporation of dynamic covalent bonds, such as Diels–Alder adducts, imines, and acetal linkages—enables the development of reprocessable materials.^4–6^

These systems, including covalent adaptable networks and dynamic polymers, allow for closed-loop recycling through thermally activated reversible mechanisms, avoiding irreversible chain degradation and maintaining mechanical performance.

However, while these design-for-recyclability routes address the issue at the synthesis stage, thermochemical conversion through catalytic pyrolysis remains a robust and necessary approach for the valorization of current heterogeneous waste streams. This process, particularly for plastics like polystyrene, yields aromatic-rich products through radical pathways involving β-scission and aromatization.^7–9^ From a mechanistic perspective, while dynamic covalent materials rely on bond exchange processes,^10,11^ pyrolysis utilizes homolytic cleavage (C–C and C–H) to convert waste into value-added chemicals. Within this framework of a circular polymer economy, understanding the surface interactions in catalytic systems is essential to optimize these end-of-life pathways.

Plastic waste, such as polyethylene (PE), polypropylene (PP), and polystyrene (PS), are suitable materials for producing fuels or high-value-added chemical feedstocks through a catalytic pyrolysis process. The use of catalysts could selectively convert plastic waste into aromatic compounds such as benzene, toluene, ethylbenzene, xylene, and naphthalenes. These chemical compounds are widely used as raw materials, solvents, and additives in the chemical, pharmaceutical, cosmetic, transportation, and various other industries.^12^

A catalyst with potential properties for the catalytic pyrolysis of plastics is cerium dioxide^13,14^ (CeO_2_) which exhibits exceptional redox properties associated with the Ce^3+^/Ce^4+^ redox couple. In addition, the surface of this material, presents oxygen vacancies and acidic and basic sites intimately related to this redox couple. These characteristics allow the ceria to catalyze numerous reactions such as dehydration, ketonization, hydrogenation, oxidation and the Suzuki–Miyaura (SM) coupling reaction.^15^ Moreover, CeO_2_ presents important properties when used as a support for metal particles, due to its peculiar nature, it has a strong influence on metal dispersion, generating strong metal–CeO_2_ interactions.^16^

The growing environmental burden posed by plastic waste, coupled with the limited effectiveness of traditional mechanical recycling, has accelerated the development of alternative valorization strategies such as chemical upcycling. Among these, pyrolysis has emerged as a particularly promising thermochemical route for converting diverse post-consumer plastics into high-value-added products, including monomers, fuels, and specialty chemicals.^2^ Unlike mechanical recycling, which often leads to downcycled materials with diminished performance, advanced recycling technologies like catalytic pyrolysis offer a pathway to close the loop by producing feedstocks suitable for new polymer synthesis or direct use in the petrochemical sector.^17^ In Argentina, initiatives such as those promoted by Ecoplas highlight the increasing integration of advanced recycling methods within circular economy models, aiming to reduce greenhouse gas emissions and optimize resource recovery from complex plastic waste streams.^3^

Much research has been carried out to infer, through experimental studies, the possible reaction mechanisms governing catalytic pyrolysis processes. Currently, computational theoretical methods are widely applied in multiple areas of chemistry and catalysis, which provide an alternative tool for understanding catalytic reactions at the molecular level. The application of methods based on density functional theory (DFT) allows the study of the reactivity of catalysts.

Using DFT simulations, the interaction of molecules on oxide surfaces,^18,19^ systems such as nanotubes and nanowires,^20,21^ intermetallic alloys,^22^ have been studied, calculating the adsorption geometries, electronic structure, charge distribution and bonding. The adsorption and dissociation of molecules on monocrystalline surfaces has also been studied, determining the molecular and electronic structure of the adsorbed species, the changes they generate on the surface and calculating dissociation barriers and vibration frequencies, obtaining a good correlation with experimental results.^23–26^

In this work, a laboratory-scale pyrolysis reactor was designed and constructed to study the catalytic conversion of polystyrene (PS) waste. The catalysts CeO_2_ and Co/CeO_2_ were synthesized and characterized by X-ray diffraction (XRD), BET surface area analysis, and surface acidity measurements. These materials were then evaluated in the catalytic pyrolysis of PS, and the resulting gas, liquid, and solid fractions were quantified by mass balance. The liquid products were further analyzed by GC-MS to determine their chemical composition. The calorific power of each liquid was determined by calorimetry.

From a theoretical approach, a CeO_2_ catalyst model was studied using self-consistent density functional theory (DFT) calculations to analyze the adsorption and subsequent dissociation of a styrene dimer on a CeO_2_ surface. Additionally, reaction pathways for the dimer dissociation on these surfaces were examined. Furthermore, cobalt was incorporated into the catalyst (Co/CeO_2_) to explore the functionalization-induced effects.

## Methodology

### Experimental characterization

The composition of the initial plastic was determined by FTIR spectroscopy using a Thermo Scientific Nicolet iS50 FTIR-NIR instrument, in the spectral range between 400 and 4000 cm^−1^. The samples were analyzed *in situ* using the ATR module.

A laboratory-scale pyrolysis reactor was designed and assembled, consisting of a heating module and a condensation module. At one end of the reactor, the plastic (approximately 2 g of PS) and nitrogen gas (10 mL min^−1^) are introduced, while a reducer containing the catalyst (200 mg) is placed at the opposite end. This configuration ensures that the gases produced during the thermal degradation of the plastic pass through the catalyst before reaching the condensation module. The operating temperature was set at 450 °C. In all cases, the yields of the three resulting products, gas, solid, and liquid, were calculated by mass balance.

The compositions of the liquids obtained from the pyrolysis reactions were analyzed by gas chromatography coupled with a mass selective detector. For this purpose, an Agilent GC 7890B gas chromatograph coupled to a 5977A mass selective detector was used. The styrene monomer percentage was determined from the relative integrated peak areas obtained from the GC-MS chromatographic profile of the pyrolysis liquid fraction. The styrene content was calculated as the ratio between the area of the styrene peak and the total integrated area of the detected liquid products. The calorific power was determined using an adiabatic bomb calorimeter (GALLENKAMP CB-200, SN: G-1877) and an analytical balance (PRECISA 125 A, Swiss Quality). This evaluation allowed us to estimate the energy capacity of the pyrolysis oils and assess their feasibility for reintegration into existing energy chains, thereby contributing to the energetic valorization of polystyrene waste.

The catalyst, CeO_2_, was synthesized using the combustion method.^27^ The synthesis was carried out using cerium nitrate hexahydrate (Sigma-Aldrich) and sucrose in a ratio of 1 mol of sucrose per mol of the desired oxide. The incorporation of Co onto the ceria surface was carried out by the wet impregnation method, followed by the appropriate thermal treatment. The catalysts used were characterized by X-ray diffraction (XRD), BET surface area, and surface acidity. X-ray diffraction experiments were performed using a Panalytical Empyrean diffractometer with CuKα_1_ radiation (*λ* = 0.15406 nm), within the 2*θ* range of 15–80°. Specific surface areas (SBET) were calculated using the BET equation. Prior to measurements, the solids were dried for 4 hours at 120 °C.

Potentiometric titrations were carried out to measure the acidity of the catalysts in an AT500N Automatic Potentiometric titrator (Kyoto instrument) equipped with an M-272 Platinum electrode (Kyoto Electronics, KEM). A 0.1 M solution of *n*-butylamine in acetonitrile was used in the titration of the suspension of 0.10 g of catalyst in 90 mL of acetonitrile. The electrode potential measured 3 h after adding 0.05 mL of the titration solution (*E*0) was taken the measurement of the strength of the acid sites, while the number of such sites was calculated from the equivalents of *n*-butylamine added till a constant potential was achieved.^26^

### Computational method

Calculations were performed using density functional theory (DFT), implemented by the Vienna *ab initio* simulation package (VASP), which uses a plane-wave basis set and a periodic supercell method.^28,29^ The electron projector augmented wave method (PAW)^30^ was employed and the spin-polarized generalized gradient approximation (GGA) with the Perdew–Burke–Ernzerhof (PBE) functional were used.^31,32^ Geometry optimizations were obtained by minimizing the total energy of the supercell using a conjugated gradient algorithm to relax the ions.^33^

Cerium dioxide catalysts were modeled considering clean and Co-modified (111) CeO_2_ surfaces. For all calculations, a kinetic energy cutoff of 400 eV was employed and the strong electron correlation effects of the Ce 4f electrons were described by a Hubbard-type on-site Coulomb repulsion using the DFT + *U* Duradev's approach with an effective *U* value of 5 eV.^34^ The surfaces were modeled using slabs of 13.5 Å by 11.7 Å with nine layers thicknesses (about 7.9 Å width), and a vacuum spacing between two repeated slabs of about 20 Å. A set of 3 × 3 × 1 Monkhorst–Pack *k*-points was used.

To study the complex process of depolymerization into styrene monomers, we developed a simplified model focused on the reactivity of a styrene dimer. First, the adsorption of a styrene dimer was investigated on the surfaces. During optimization, adsorbed species and the first two surface layers were allowed to relax. Also, for all the geometry optimizations an energy cut condition of 10^−2^ eV for the total energy of the system between two ionic relaxation steps, was considered. The electronic relaxation convergence criterion was set to 10^−3^ eV. van der Waals interaction between pairs was included by means of Grimme DFT-D2 method.^35^

Dimer dissociation to styrene monomers was also investigated. We modeled possible reaction pathways using the climbing image nudged elastic band method (CI-NEB).^36^ Nine geometry images for each system were considered. The nature of the transition states on the potential energy surface has been tested based on the analysis proposed by Henkelman *et al.*^37^ and its configuration was verified by a vibrational analysis, finding one imaginary frequency.

Total density of states (DOS) and projected density of states (PDOS) curves were used to analyze the electronic structure. The electronic charges on atoms were computed using Bader analysis.^38^

## Results and discussion

### Experimental characterization

The FTIR spectrum of polystyrene exhibits several characteristic absorption bands associated with its aromatic and aliphatic structure. The region between 3083–3025 cm^−1^ corresponds to the stretching vibrations of aromatic C–H bonds, indicating the presence of phenyl rings. The 2920–2850 cm^−1^ bands are attributed to the aliphatic C–H stretching vibrations from the polymer backbone, reflecting the saturated hydrocarbon chains in the styrene units.

Strong peaks at 1600, 1493, and 1452 cm^−1^ are due to the C=C stretching and C–H bending vibrations within the aromatic rings, confirming the polystyrene's rigid phenyl group structure. The 1028–1068 cm^−1^ bands are associated with in-plane bending of aromatic C–H bonds, while the distinctive out-of-plane bending vibrations at 758 and 698 cm^−1^ are characteristic of monosubstituted benzene rings, which are a defining feature of polystyrene.

Overall, the presence and intensity of these bands confirm the identity of the polymer as polystyrene and provide insight into its molecular structure, with strong aromatic character and a saturated hydrocarbon backbone (Table 1).

**Table 1:** The FTIR spectrum of polystyrene waste

Wavenumber (cm^−1^)	Vibrational mode	Assignment
3083–3025	C–H stretching (aromatic)	Aromatic ring C–H stretching
2920–2850	C–H stretching (aliphatic)	Aliphatic C–H from polymer backbone
1600	C=C stretching (aromatic)	Aromatic ring skeletal vibration
1493	C=C stretching (aromatic)	Aromatic ring vibration
1452	C–H bending (aliphatic)	CH_2_/CH_3_ deformation
1068–1028	C–H in-plane bending (aromatic)	In-plane deformation of aromatic C–H
758	C–H out-of-plane bending (aromatic)	Monosubstituted benzene ring
698	C–H out-of-plane bending (aromatic)	Monosubstituted benzene ring

**Fig. 1:**
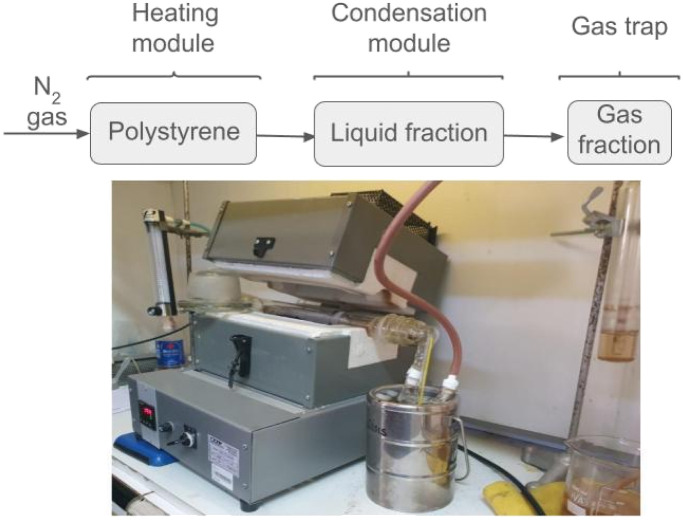
Schematic diagram of the pyrolysis reactor.

A pyrolysis reactor was constructed (Fig. 1), consisting of a heating module and a condensation unit. Polystyrene and a nitrogen stream are fed into one end of the heating module, while at the opposite end, within a narrowing section, the catalyst is placed. This configuration ensures that the pyrolysis vapors pass through the catalytic bed before reaching the condenser. Thermal pyrolysis reactions of polystyrene were conducted to optimize the reaction temperature, evaluating 400, 450, and 500 °C, with the aim of maximizing the yield of pyrolytic liquids. Additionally, catalytic pyrolysis experiments were carried out using CeO_2_ and Co/CeO_2_ as catalysts.

**Fig. 2:**
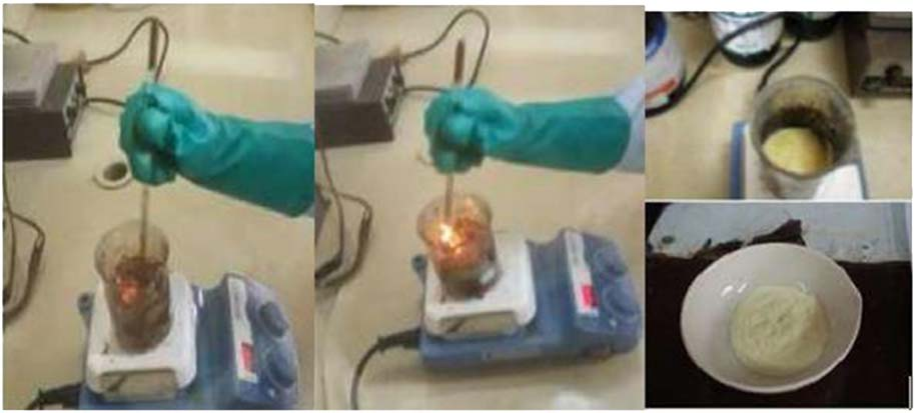
Synthesis of CeO_2_.

To obtain CeO_2_, the combustion synthesis was carried out using hexahydrated cerium nitrate (Sigma Aldrich) and sucrose, which were dissolved in the minimum amount of water and heated on a hot plate until dryness. Combustion occurred spontaneously, initiating self-ignition with a visible flame. During the oxide formation, nitrous vapors and CO_2_ were released, as shown in Fig. 2. If an excess amount of sucrose is added, a very violent reaction takes place. To eliminate carbonaceous residues, the resulting material was calcined in a muffle furnace at 400 °C for 2 hours.

The reaction that takes place during the formation of cerium oxide by combustion is as follows:Ce(NO_3_)_2(aq)_ + C_12_H_22_O_11(aq)_ + *y*O_2(g)_ → CeO_2(s)_ + 2N_2(g)_ + 2NO_*x*(g)_ + 12CO_2(g)_ + 11H_2_O_(g)_where *y* = 5 + *x*.

**Fig. 3:**
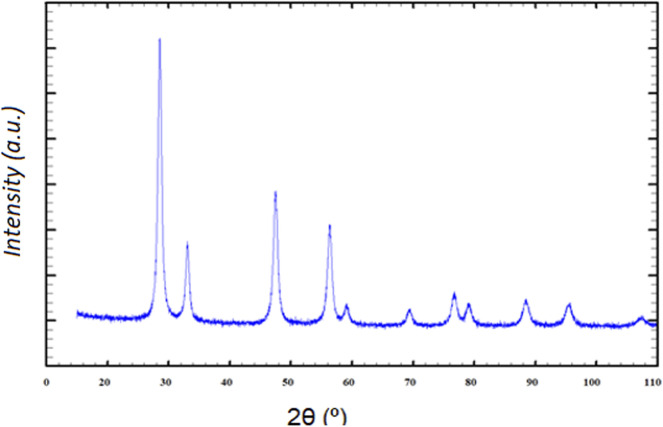
XRD pattern of CeO_2_.

The material obtained through the synthesis method used for the preparation of CeO_2_ exhibits a diffraction pattern characteristic of the face-centered cubic fluorite-type structure, corresponding to space group *Fm*3*m*, according to JCPDS card no. 34-0394. Fig. 3 shows the absence of peaks attributable to secondary phases indicates a high purity of the synthesized cerium oxide.

The Co/CeO_2_ catalyst was prepared by wet impregnation of combustion-synthesized ceria, followed by thermal treatment. The X-ray diffraction pattern of the Co/CeO_2_ material was essentially identical to that of bare CeO_2_, indicating that the incorporation of cobalt did not induce detectable changes in the fluorite crystal structure of the support. Moreover, no additional diffraction peaks attributable to crystalline cobalt oxide phases were observed. This lack of segregated cobalt-related reflections suggests that cobalt is highly dispersed on the ceria surface, likely as isolated surface species or finely distributed nanometric domains below the detection limit of XRD.

The pyrolysis reactor shown in Fig. 1 was used to study the catalytic conversion of polystyrene (PS) waste. The optimal operating conditions, under which the liquid product yields were maximized, were determined to be: *T* = 450 °C, plastic waste mass = 2 g, N_2_ flow rate = 20 mL min^−1^, and reaction time = 5 min, with an optimal plastic waste-to-catalyst mass ratio of 10.

The performance of CeO_2_, synthesized *via* the combustion method, was evaluated as a catalyst for the pyrolysis of polystyrene (PS) and compared to a non-catalyzed reaction. See Table 2. The parameters analyzed included specific surface area (SBET), liquid yield, styrene selectivity, and the calorific value of the resulting pyrolytic oil. The CeO_2_ and Co/CeO_2_ catalysts exhibited a high specific surface area (110 and 100 m^2^ g^−1^ respectively), which enhances the interaction between PS chains and the active sites, thereby promoting selective reactions.

**Table 2:** Major Components of the Liquids from PS Pyrolysis

Catalyst	SBET (m^2^ g^−1^)	Liquid fraction yield (%)	Styrene (%)	Calorific value (kJ kg^−1^)
PS without catalyst	—	46.24	60.66	42.88
CeO_2_	110	20.00	87.04	34.54
Co/CeO_2_	100	37.00	60.08	41.34

The catalytic pyrolysis of polystyrene (PS) showed marked variations in both product distribution and quality depending on the catalyst used. Under non-catalytic conditions, the process produced the highest liquid yield (46.24%) and calorific value (42.88 kJ kg^−1^), with a styrene content of 60.66%, characteristic of a purely thermal degradation pathway. When CeO_2_ was employed as a catalyst, despite its high specific surface area (110 m^2^ g^−1^), the liquid yield decreased significantly to 20.00%, as a considerable portion of the pyrolysis vapors adhered to the catalyst surface. Nonetheless, CeO_2_ exhibited outstanding selectivity toward styrene (87.04%), highlighting its potential for monomer recovery. The calorific value of the liquid fraction decreased to 34.54 kJ kg^−1^, indicating a compositional shift toward lighter and less energy-dense hydrocarbons.

Pyrolysis oils are commonly blended with conventional fuels; however, when the pyrolysis liquid is rich in styrene monomer, its heating value decreases considerably. In such cases, this liquid, after the separation of the predominant monomeric component, could instead be used for the synthesis of second-generation polystyrene, thereby illustrating the upcycling concept.

In contrast, the bimetallic Co/CeO_2_ catalyst offers a more balanced performance. Despite a slightly lower surface area (100 m^2^ g^−1^), it increases the liquid fraction yield to 37.00%, indicating lower interaction between the pyrolysis vapors and the catalyst surface. The styrene content (60.08%) is comparable to that of non-catalytic pyrolysis, but with a notable improvement in energy density, maintaining a combustion value similar to that of the uncatalyzed oil (41.34 kJ kg^−1^). These results suggest that the use of the Co/CeO_2_ catalyst preserves the thermal degradation pathway and partially maintains the formation of target monomers such as styrene.

CeO_2_ has proven to be an efficient catalyst for the selective depolymerization of polystyrene (PS) into styrene monomer, primarily due to its acidic surface, which promotes a β-scission mechanism. In contrast, the addition of cobalt species to the CeO_2_ support alters the reaction pathway, resulting in a lower styrene yield but improving overall liquid product yield and energy content. This suggests that Co/CeO_2_ offers a favorable balance between monomer recovery and fuel-grade product generation, positioning it as a versatile catalyst for both material and energy recovery from PS waste. According to Park and colleagues,^39^ β-scission at the chain end of PS is the predominant mechanism responsible for styrene formation, highlighting the crucial role of catalyst surface properties in directing product distribution.

However, if the objective is to promote styrene monomer formation, the incorporation of cobalt onto the surface of CeO_2_ appears to hinder the interaction of pyrolysis vapors with the acidic sites of cerium oxide, thus preventing the catalytic process from being directed toward styrene monomer production. In this sense, the cobalt acts by altering the pristine CeO_2_ surface, thereby modifying the styrene monomer yields.

### Theoretical calculations

We modeled two cerium dioxide catalysts, starting with a pristine CeO_2_ (111) surface. This surface was based on a face-centered cubic fluorite-type structure, which aligns with the spectral data from our measured samples. On this pristine surface, charge analysis revealed that surface oxygen atoms possess a negative charge of −1.19*e* and subsurface cerium atoms have a positive charge of 2.37*e*.

**Fig. 4:**
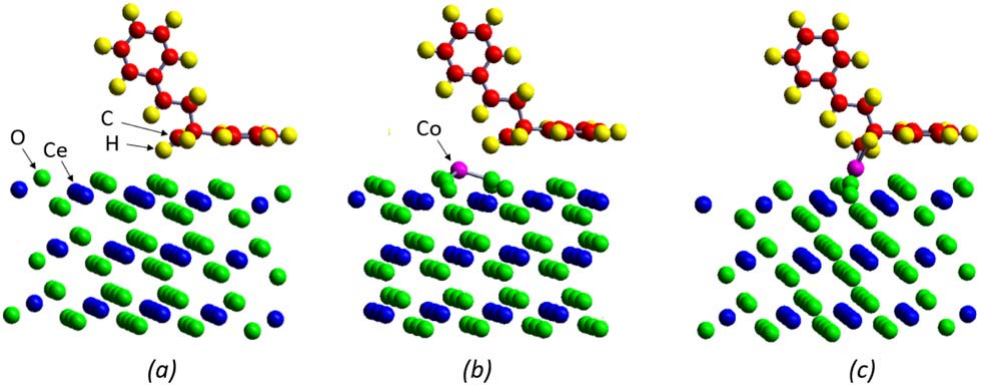
Surface structure of styrene dimer adsorbed on O top site of CeO_2_ (a), on O top site of Co–CeO_2_ (b) and on Co top site of Co–CeO_2_ (c).

To represent the experimental observations described above, a Co-modified CeO_2_ (111) surface was analyzed (both surface models are shown on Fig. 4). For this, pre-adsorbed cobalt at threefold positions was considered on the surface (several Co locations were computed, being the three-fold oxygen sites the most stable ones). The optimized average Co–O distance was determined to be 1.93 Å. The adsorption also caused a displacement of the nearest-neighbor oxygen atoms by 0.3 Å away from the surface. The charge analysis revealed that the Co atom possesses a positive charge of 1.18*e*, and the charge of its nearest-neighbor oxygen atoms increased by 3–4%. The net effect is a positive charged surface.

The adsorption of a styrene dimer was investigated on both catalyst models. On the pristine CeO_2_(111) surface, the most stable configuration was found to involve a hydrogen atom from the CH_2_ group forming a bond with a top-site oxygen atom (O site). This configuration, as depicted in Fig. 4a, has an adsorption energy of −0.49 eV and an O–H bond length of 2.07 Å. Intermolecular distances and angles exhibit no significant structural deviations following the adsorption process.

On the Co-modified surface, we analyzed the adsorption of the dimer at a top oxygen site near the Co atom (O site), a configuration similar to that found on the pristine surface (Fig. 4b). The resulting O–H bond length of 2.27 Å is almost 10% longer, leading to a less favorable configuration with lower adsorption energy of −0.06 eV.

In contrast, a much stronger adsorption was observed at a Co top site (Co site), exhibiting an adsorption energy of −0.82 eV (Fig. 4c). In this configuration, the carbon atom from the CH_2_ group bonds with the Co atom, resulting in Co–C bond length of 1.88 Å. This shorter dimer–surface distance, relative to the pristine case, reflects a stronger adsorption interaction. In these cases, as well, structural deviations are minimal, indicating that the molecule undergoes no substantial deformation upon adsorption.

After adsorption on the pristine surface the dimer retains a near-neutral charge, while the whole surface has a small positive charge of +0.084*e* (this is the total charge of the stoichiometric CeO_2_ surface layer), see Table 3.

**Table 3:** Net charges in electron units

Charge (e)	C_16_H_16_/CeO_2_	C_16_H_16_/Co–CeO_2_	
		O Site	Co site
Dimer	0.091	0.084	−0.102
Upper ring	0.090	0.053	0.103
Lower ring	0.001	0.031	−0.205
Surface	0.084	0.224	0.376
C^a^	−0.171	−0.094	−0.260
Co^b^	—	1.174	0.853

a From CH_2_ group, as indicated with an arrow in Fig. 4a.

b Co charge on isolated surface is +1.180*e*.

When the dimer adsorbs onto an oxygen site on the Co-modified surface (a site analogous to that on the pristine surface), it remains nearly neutral, though the surface positive charge increases to +0.224*e*. In a distinct scenario, adsorption onto a Co-top site results in the dimer becoming negatively charged. This charge is distributed such that the ring parallel to the surface (lower ring, in Table 3) acquires a negative charge (with a notable increase in the negative charge of the C atom bonded to Co), while the ring farther from the surface (upper ring) becomes positive. This configuration leads to a larger positive surface charge of +0.376*e*. Consistent with the calculated adsorption energy, this latter configuration exhibits a stronger adsorption than the previously discussed cases.

**Fig. 5:**
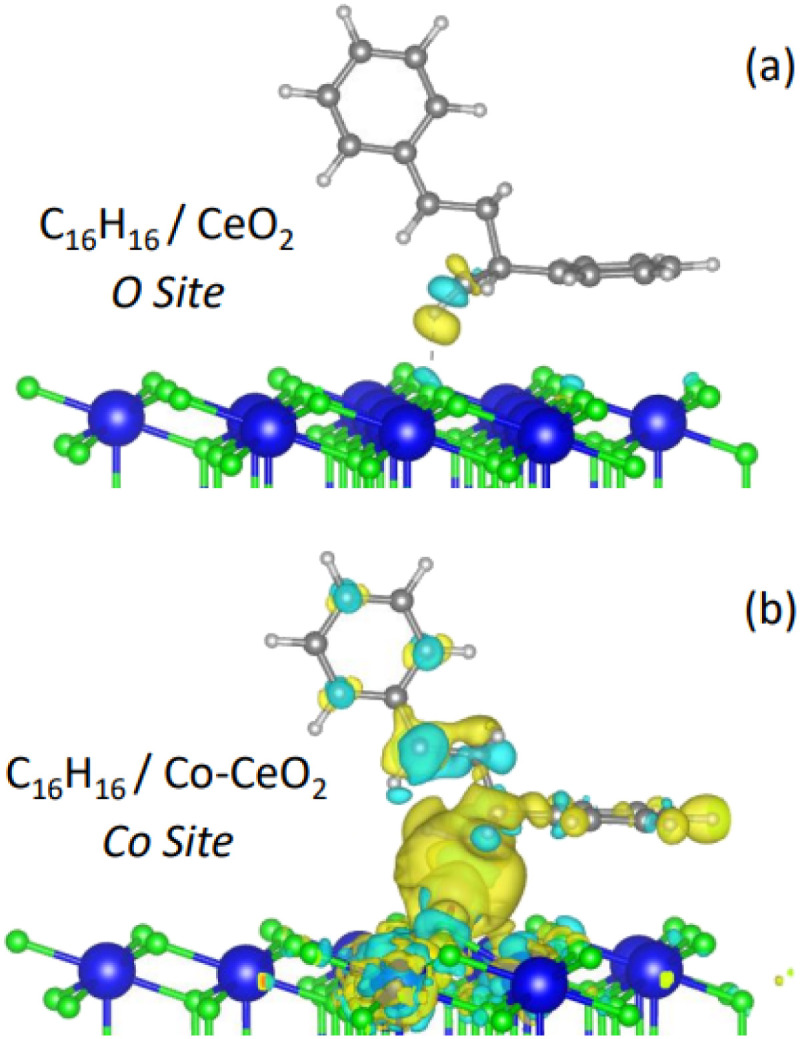
Charge density difference plots for a dimer adsorbed on (a) pristine CeO_2_ and (b) a Co top site of the Co–CeO_2_ surface. Yellow regions represent charge accumulation (negative), while light blue regions indicate charge depletion (positive).

Fig. 5 shows the plot of the charge density for the dimer adsorbed on pristine surface (a) and on a Co top site on the Co-modified surface (b). The charge density difference (Δ*ρ*) isosurface is calculated using the following equationΔ*ρ* = *ρ* (dimer/surface) − *ρ* (surface) − *ρ* (dimer)where *ρ* (dimer/surface) is the charge density of the dimer on the surface *ρ* (surface) is that of the surface after adsorption but without the adsorbate and *ρ* (dimer) is that of the dimer in the adsorption geometry, but without the substrate.

Fig. 5a illustrates the nearly neutral character of the dimer when adsorbed on the pristine surface. In contrast, when positioned on a Co top site (Fig. 5b), there is a visible accumulation of negative charge around the lower ring (yellow), while the upper ring exhibits charge depletion (light blue). This qualitative observation is in good agreement with the Bader charge analysis.

**Fig. 6:**
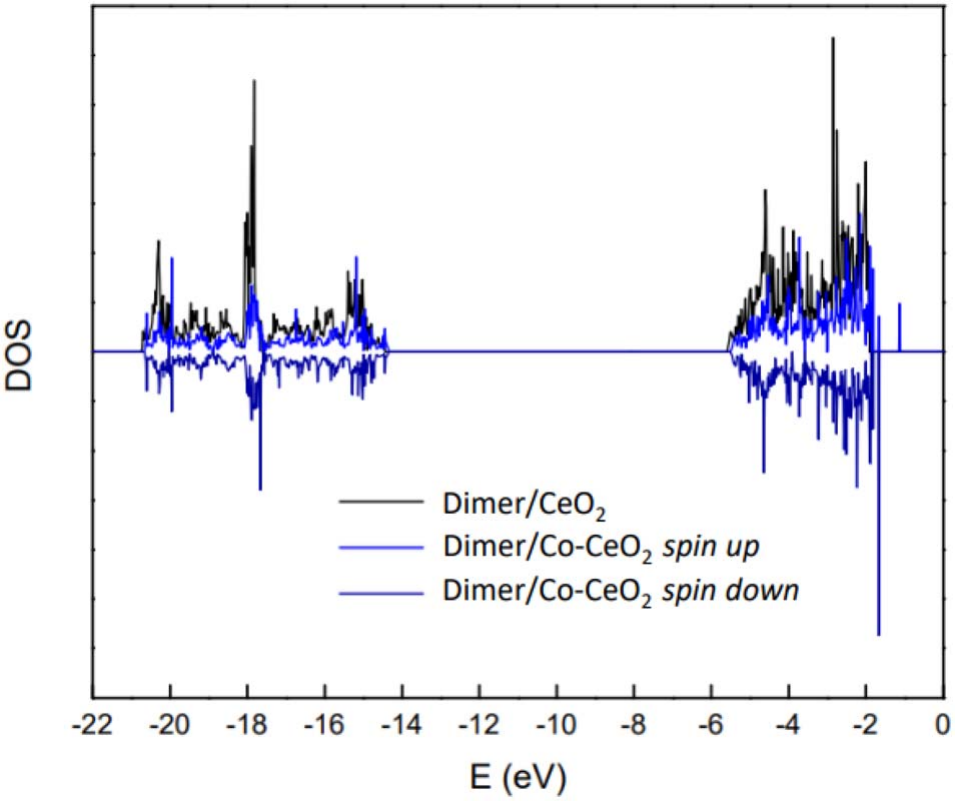
DOS curves for dimer adsorbed on CeO_2_ surface (black line) and on Co-modified surface (blue lines corresponding to spin up and down projections).

Density of States (DOS) curves were calculated for the dimer adsorbed on the bare CeO_2_ surface (black line, Fig. 6) and on a Co-top site of the Co-modified surface. Due to the magnetic nature of the Co-modified system, both spin-up and spin-down contributions are explicitly shown (blue lines, Fig. 6)

There is a wide energy range of occupied states (there is also a strong peak at −35 eV due to s surface orbitals, out of scale on these figures, which is not relevant for the present analysis). Qualitatively, the systems differ mainly in the peaks within the 0 to −2 eV range upon dimer adsorption on the Co-modified surface.

**Fig. 7:**
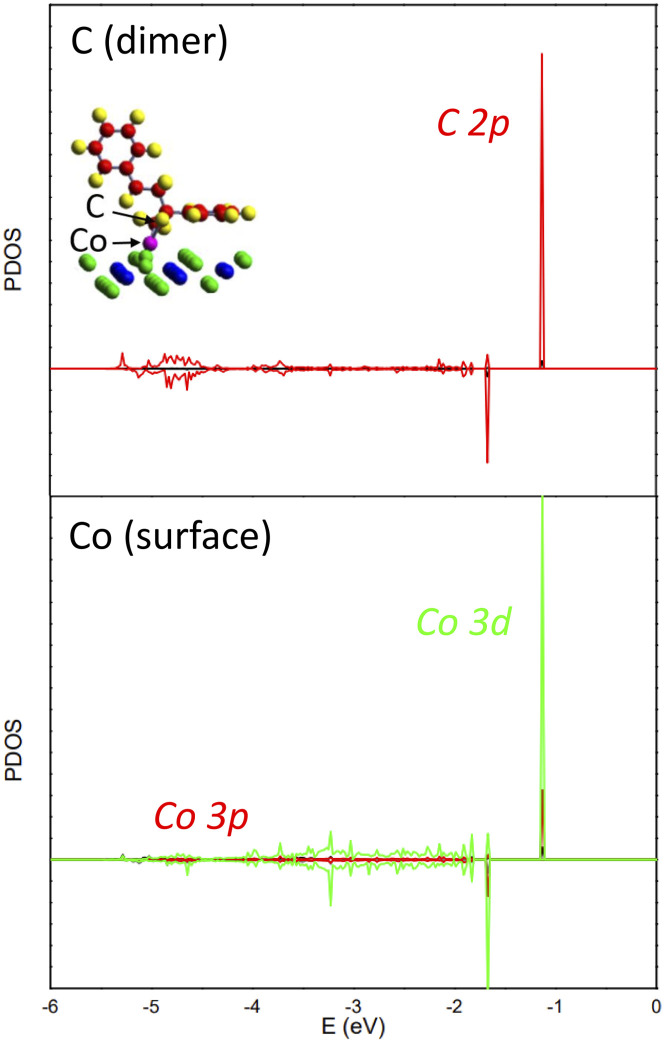
PDOS onto the atomic orbitals of C (dimer) and Co (surface) atoms. Red and green curves represent p and d states, respectively. The inset provides a schematic of the system, illustrating the positions of the indicated atoms.

To clarify the bonding mechanism, we calculated the PDOS by projecting states onto site-centered atomic orbitals. The PDOS for Co and the dimer's C atom (Fig. 7) shows new peaks at about −1.0 and −1.7 eV. These are mainly derived from C 2p and Co 3d interactions, providing evidence for the strong Co–C coupling observed.

The dissociation of the styrene dimer into two monomeric units was investigated. Initially, the dimer was adsorbed onto a top site of the surface, as previously described. During the dissociation process, the upper monomer (positioned farther from the surface) detached and moved away, while the lower monomer remained anchored to the surface. This process occurred through cleavage of a C–C bond, likely *via* a β-scission mechanism.

**Fig. 8:**
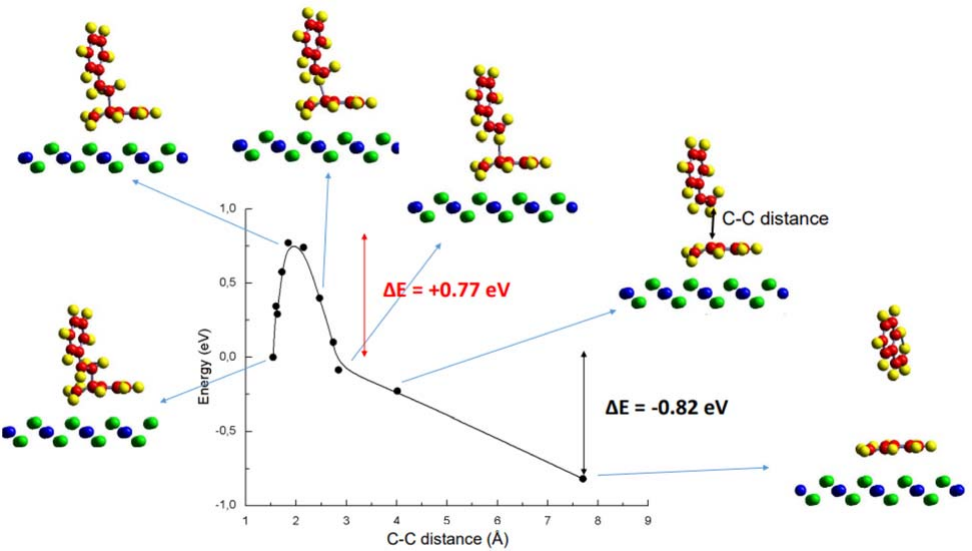
Reaction coordinate for the dissociation of a styrene dimer adsorbed on the pristine CeO_2_ surface, showing the energy variation during the transition to two styrene monomer units.

The reaction pathway from the initial adsorbed dimer to the dissociated state was investigated using CI-NEB (Climbing Image Nudged Elastic Band) calculations. Fig. 8 illustrates the energy curve as a function of the C–C bond distance, which serves as the reaction coordinate. Geometry corresponding to selected points along the path are also shown. The energy of the initial adsorbed dimer is taken as the zero-energy reference. The dissociation of the dimer is an energetically favourable process: upon dissociation, the energy of the system decreased by 0.82 eV, and an activation energy barrier of 0.77 eV was observed. Another reaction pathway, leading to a final state with both monomers adsorbed on the surface, was also explored. This path was found to exhibit higher energy barriers.

**Fig. 9:**
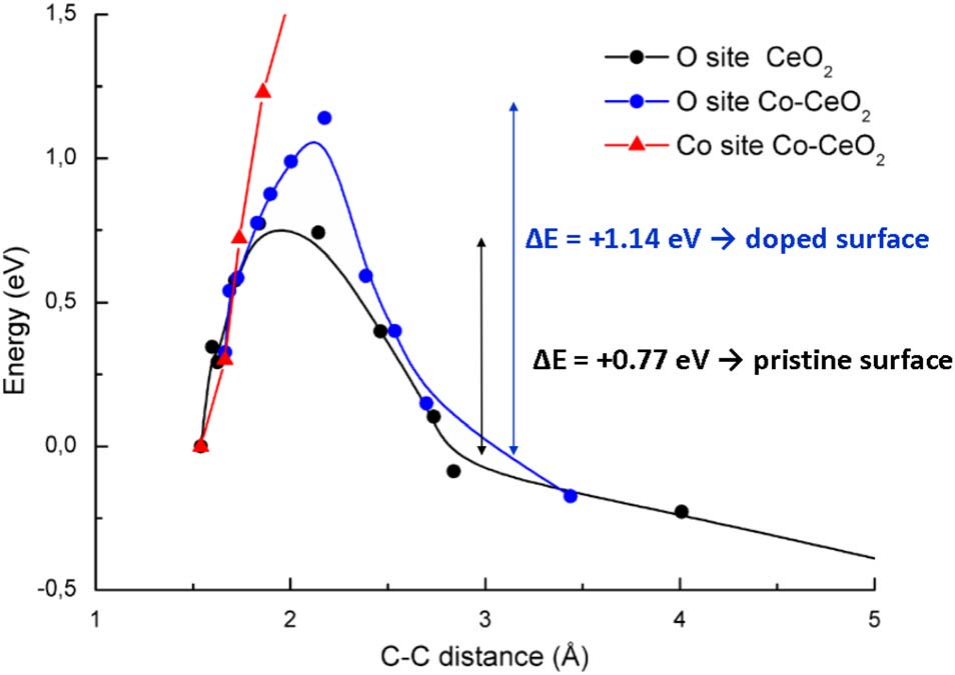
Reaction coordinate for the dissociation of a styrene dimer initially adsorbed on the pristine CeO_2_ surface (black line), and on the Co-modified surface on a O top site (blue) and on a Co top site (red).

Analogous reaction pathways were also investigated on the Co-modified surface. When the styrene dimer is adsorbed at a top oxygen site adjacent to a cobalt atom, the results are qualitatively comparable to those observed on the pristine surface. However, the activation barrier is higher on the Co-modified surface, reaching 1.14 eV (blue curve, Fig. 9). In contrast, direct adsorption of the dimer onto a cobalt atom was found to be energetically unfavourable for dissociation. The separation of the dimer on this site leads to a substantial increase in energy, indicating that this process is not a viable reaction pathway (red curve, Fig. 9).

## Conclusions

The experimental study confirmed the successful synthesis and characterization of CeO_2_ and Co/CeO_2_ catalysts, as well as the efficient operation of a laboratory-scale pyrolysis reactor designed for the conversion of polystyrene (PS) waste. FTIR analysis of the PS feedstock validated its aromatic structure, while XRD characterization of the synthesized catalysts confirmed the formation of a pure fluorite-type CeO_2_ phase and the high dispersion of cobalt on its surface. The optimization of operational parameters (*T* = 450 °C, N_2_ flow rate = 20 mL min^−1^, reaction time = 5 min, and plastic-to-catalyst mass ratio = 10) enabled maximizing liquid product yields.

The results have shown that catalyst composition plays a decisive role in controlling product selectivity and yield. Pure CeO_2_, with its high surface area and acidic sites, promoted selective depolymerization of PS toward styrene monomer formation through a β-scission mechanism. In contrast, the incorporation of cobalt onto the CeO_2_ surface modified the reaction pathway, reducing styrene selectivity but increasing overall liquid yield and energy content. These findings indicate that CeO_2_ is suitable for processes aimed at monomer recovery, whereas Co/CeO_2_ represents a more balanced catalyst for both energy and material valorization of PS waste. The study highlights the importance of catalyst surface properties in directing pyrolysis reactions and provides a foundation for optimizing catalytic systems for selective chemical recycling of plastics.

Our theoretical calculations suggest that the styrene dimer adsorbs on CeO_2_ surfaces and its subsequent dissociation to a gas-phase styrene monomer *via* a β-scission mechanism is energetically favorable. These findings are consistent with our experimental results for the CeO_2_ catalyst.

Moreover, the presence of cobalt elevates the activation barrier for dissociation. This finding is in good agreement with experimental results showing a decrease in styrene yield on cobalt-modified catalysts. Additionally, cobalt incorporation creates sites where the styrene dimer adsorbs so strongly that it does not dissociate but remains permanently bound to the surface. This proposed mechanism provides a molecular-level explanation for the observed yields of styrene monomer.

## Author contributions

Dana Yanes Quintana: investigation, Victoria Colombo: investigation, Maria Estela Pronsato: formal analysis Nilda Chasvin: investigation, formal analysis, writing – review & editing, Carolina Pistonesi: investigation, formal analysis writing – review & editing and Alejandra Diez: investigation, writing – review & editing.

## Conflicts of interest

The authors declare no conflict of interest.

## Data Availability

The data that support the findings of this study are available from the corresponding author upon reasonable request.
